# Aromatase Inhibitor Therapy Is Associated with Distinct Plasma Lipidomic Profiles in Postmenopausal Breast Cancer Patients

**DOI:** 10.3390/ijms27041926

**Published:** 2026-02-17

**Authors:** Aleksandra Arsic, Ales Kvasnicka, David Friedecky, Nebojsa Ivanovic, Maja Milosevic, Vesna Vucic

**Affiliations:** 1 Group for Nutritional Biochemistry and Dietology, Institute for Medical Research, National Institute of Republic of Serbia, University of Belgrade, 11000 Belgrade, Serbia; mmilosevic@imi.bg.ac.rs (M.M.); vesna.vucic@imi.bg.ac.rs (V.V.); 2 Laboratory for Inherited Metabolic Disorders, Department of Clinical Biochemistry, University Hospital Olomouc and Faculty of Medicine and Dentistry, Palacký University Olomouc, 779 00 Olomouc, Czech Republic; alkvas@ous-hf.no (A.K.); david.friedecky@fnol.cz (D.F.); 3 Department of Medical Biochemistry, Oslo University Hospital, 0372 Oslo, Norway; 4 Faculty of Medicine, University of Belgrade, 11000 Belgrade, Serbia; ivanovicnebojsadr@gmail.com; 5 Department of Surgical Oncology, University Hospital Medical Center “Bezanijska Kosa”, 11000 Belgrade, Serbia

**Keywords:** lipidomics, breast cancer, aromatase inhibitor therapy, sphingolipid metabolism

## Abstract

Aromatase inhibitors (AIs) are the standard adjuvant endocrine therapy for postmenopausal women with hormone receptor-positive breast cancer; however, their effects on lipid metabolism remain incompletely characterized. In this study, we investigated AI-associated alterations in the plasma lipidome using mass spectrometry-based lipidomics. Plasma samples were collected from 30 patients prior to AI initiation and 29 patients receiving non-steroidal AI therapy for at least 24 months. Ultra-high-performance liquid chromatography–tandem mass spectrometry identified and relatively quantified 649 lipid species across 23 lipid classes and subclasses. Lipidomic analysis revealed significant differences in specific lipid species. Several phosphatidylcholine, sphingomyelin, and lysophosphatidylethanolamine species were significantly more abundant in patient plasma prior to AI therapy, whereas higher levels of selected ceramides, hexosylceramides, phosphatidylinositol (PI 16:0_16:0), and a polyunsaturated diacylglycerol species were observed in patients receiving AI therapy. Multivariate analyses revealed patient group separation, and a Naive Bayes classification model based on lipid-class levels achieved an area under the curve of 0.79. Additionally, lipid network and hierarchical clustering analyses identified systematic lipid-class trends. Protein–protein interaction network analysis based on lipidomic profiles highlighted enzymes associated with sphingolipid metabolism pathways. These findings demonstrate that long-term AI therapy is associated with specific alterations in the plasma lipidome, consistent with estrogen-deprivation-related metabolic differences. Targeted lipidomic profiling may provide mechanistic insights into therapy-associated metabolic effects and support future efforts to optimize long-term management of breast cancer survivors.

## 1. Introduction

Breast cancer (BRC) is the most common malignancy among women, with over 2.2 million new cases diagnosed globally in 2022 [1]. A substantial proportion of breast cancers are estrogen receptor-positive (ER+) and/or progesterone receptor-positive (PR+) [2], necessitating multimodal treatment approaches, including surgery, chemotherapy, radiotherapy, and endocrine therapy. Aromatase inhibitors (AIs) represent the gold-standard adjuvant endocrine treatment for postmenopausal women, as they improve disease control and reduce the risk of distant metastasis by inhibiting peripheral aromatase activity [3]. This inhibition prevents the conversion of androgenic precursors into estrogens in peripheral tissues, resulting in a marked reduction in systemic estrogen levels. For postmenopausal women with ER+ and PR+ breast tumors, a five-year course of AI therapy, often extended beyond this duration, is recommended to reduce the risk of disease recurrence [4].

AIs decrease plasma and tissue concentrations of endogenous estrogens by inhibiting aromatase (cytochrome P450), the rate-limiting enzyme responsible for estrogen biosynthesis from testosterone and adrenal androgens [5]. Because estrogen plays a protective role in lipid metabolism, AI therapy is frequently associated with dyslipidemia. Increased levels of triglycerides (TG), total cholesterol (TC), and low-density lipoprotein cholesterol (LDL) have been reported in patients receiving AIs [6], thereby increasing the risk of cardiovascular disease (CVD) in postmenopausal women with BRC [7].

Disturbances in lipid metabolism are recognized as a hallmark of cancer, leading to increasing interest in lipidomic profiling as a tool for characterizing tumor-associated metabolic alterations [8]. In recent years, clinical lipidomics studies have been conducted across multiple malignancies, including lung [9], pancreatic [10], renal cell carcinoma [11], colorectal [12], and BRC [13], highlighting cancer-specific lipidomic signatures and the growing relevance of lipid metabolism in oncology. Lipidomic reprogramming is now recognized as a defining feature of BRC. To date, elevated levels of phosphatidylcholine (PC 32:1), stearic acid, and ceramide (Cer 43:1), along with reduced levels of diacylglycerol (DG 34:2), have been reported in BRC patients compared with healthy women [14]. These lipid species, together with phosphatidylinositol (PI 16:0/16:1) and PI (18:0/20:4), have been proposed as potential biomarkers for BRC [14].

Despite these advances, there remains a paucity of studies investigating the lipidomic profiles in BRC patients in relation to endocrine therapy. The present cross-sectional study aims to address this gap by conducting mass spectrometry-based profiling of lipid classes and comparing profiles between postmenopausal BRC patients receiving AI therapy and another cohort assessed before this treatment. This approach aims to characterize associations between AI therapy status and plasma lipidomic patterns.

## 2. Results

### 2.1. Baseline Characteristics

This study included 30 BRC patients assessed prior to the initiation of the AI therapy (BRC group) and 29 patients who had been receiving AI therapy for at least two years (AI group). The anthropometric characteristics at recruitment and the clinical characteristics (BRC stage and subtype) at the time of diagnosis are summarized in Table 1.

### 2.2. Biochemical Parameters

The biochemical parameters of patients in both groups are presented in Table 2. No significant differences in biochemical parameters were observed between the BRC and AI groups apart from the high-density cholesterol levels.

### 2.3. Lipidomic Analysis

**Figure 1:**
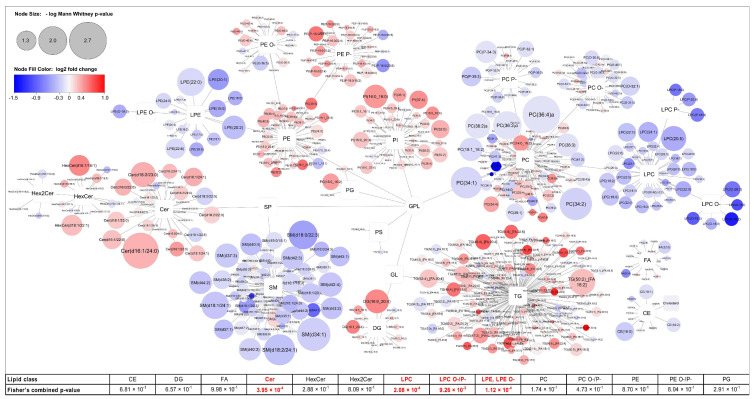
Global lipid network analysis of plasma lipidomic differences between breast cancer patients (BRC) prior to initiation of the aromatase inhibitor (AI) therapy and patients receiving AI. Cytoscape-based network visualization illustrates lipid species grouped according to structural similarity and lipid class. Each node represents an individual lipid species, while edges indicate class-based relationships. Node size corresponds to the −log10 *p*-value from the Mann–Whitney U test, reflecting the statistical significance (−log *p*-Value > 1.3) of differences between groups. Node color indicates the direction and magnitude of change, based on log2 fold change, with red representing higher abundance in AI-treated patients and blue representing higher abundance in patients before AI therapy. Major lipid classes, including glycerophospholipids (GPL), sphingolipids (SP), glycerolipids (GL), fatty acids (FA), and cholesteryl esters (CE), are shown to highlight coordinated class- and species-level lipidomic alterations. Fisher’s combined *p*-value for individual lipid classes is shown at the bottom (significant *p*-Value < 0.05).

Plasma lipid profiling identified 649 lipids across 23 lipid classes and subclasses, covering the major structural families of the circulating lipidome, including neutral lipids (triacylglycerols, diacylglycerols, cholesteryl esters, and cholesterol), glycerophospholipids (phosphatidylcholines, phosphatidylethanolamines, phosphatidylinositols, phosphatidylglycerols, phosphatidylserines, and their lysophospholipid counterparts), sphingolipids (sphingomyelins, ceramides, monohexosylceramides, and dihexosylceramides), and free fatty acids. In addition to diacyl phospholipids, the dataset included ether-linked and plasmalogen species (denoted as O- and P-), most prominently within PC, PE, and LPC and LPE classes. An overview of the plasma lipidome is shown as a global lipid network (Figure 1). To assess whether the observed lipid changes reflect coordinated remodeling rather than isolated signals, we performed Fisher’s combined probability test at the lipid-class level. This analysis revealed significant cumulative differences in several lipid classes, including Cer, LPC, ether/plasmalogen LPC, LPE, and SM (Figure 1), supporting systematic class-level alterations despite limited power at the single-species level.

### 2.4. Multivariate Analysis of Plasma Lipidomic Profiles

**Figure 2:**
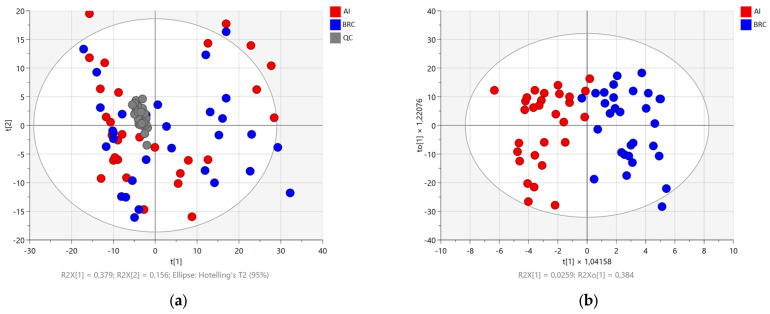
Multivariate analysis of plasma lipidomic profiles in breast cancer patients (BRC) prior to initiation of the aromatase inhibitor (AI) therapy and patients receiving AI. Principal component analysis (PCA) score plot (**a**) shows the distribution of plasma lipidomic profiles from patients before AI therapy (BRC, blue), patients receiving AI therapy (AI, red), and quality control samples (QC, gray). Orthogonal partial least squares–discriminant analysis (OPLS-DA) score plot (**b**) demonstrates improved separation between BRC and AI groups. The ellipse represents the 95% Hotelling’s T^2^ confidence interval.

Principal component analysis (PCA) and partial least squares discriminant analysis (OPLS-DA) were applied to the lipidomic dataset to assess global variation and group separation between the BRC and AI groups (Figure 2). PCA was performed as an unsupervised analysis, including all samples and quality control (QC) samples. The first two principal components explained 37.9% (PC1) and 15.6% (PC2) of the total variance, accounting for 53.5% of the cumulative variance. QC samples clustered tightly near the center of the PCA score plot, indicating analytical stability and reproducibility of the LC–MS measurements. In the PCA score plot, samples from the BRC and AI groups showed partial overlap, with no complete separation observed along the first two principal components. To further evaluate group discrimination, a supervised OPLS-DA model was constructed using the same dataset. The OPLS-DA score plot demonstrated improved separation between the BRC and AI groups along the first latent variable, with group-specific clustering highlighted by confidence ellipses. Permutation testing confirmed that the supervised OPLS-DA model was not overfitted, with a negative Q^2^ intercept (Q^2^ = −0.55), indicating that permuted models lacked predictive ability compared with the original model (Supplementary Figure S1). Together, these multivariate analyses indicate differences in lipidomic profiles between the BRC and AI groups, while also demonstrating acceptable analytical performance as evidenced by the clustering of QC samples.

### 2.5. Differential Lipid Species Between the BRC and AI Groups

**Figure 3:**
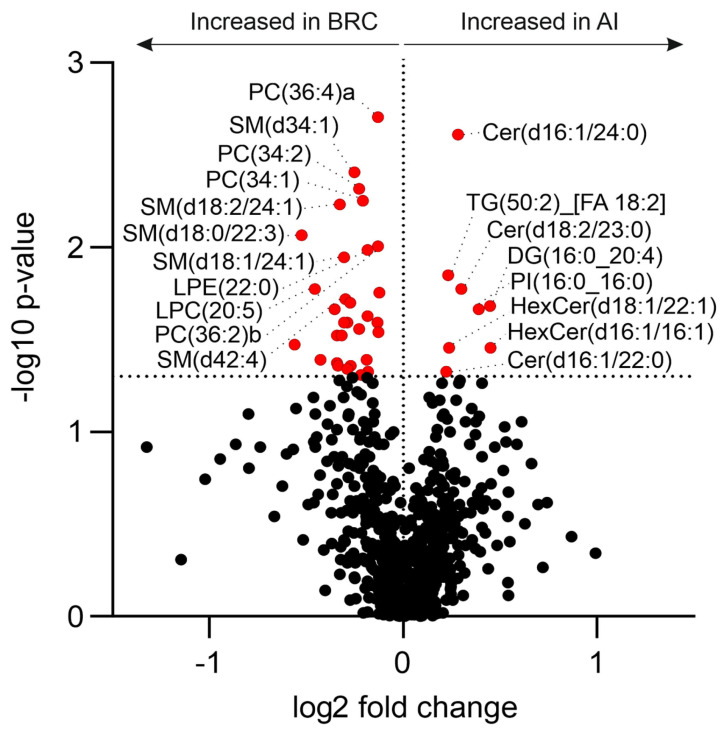
Volcano plot showing differential plasma lipid species between breast cancer patients (BRC) prior to initiation of the aromatase inhibitor (AI) therapy and patients receiving AI. The x-axis represents log_2_ fold change (AI vs. BRC), and the y-axis shows −log_10_-transformed *p*-values from the Mann–Whitney U test. The vertical dotted line indicates no change between groups, and the horizontal dotted line denotes the statistical significance threshold (*p*-Value < 0.05). Lipid species significantly increased in the BRC group are shown on the left, while those increased in the AI group are shown on the right. Red dots represent statistically significant lipid species, whereas black dots indicate non-significant lipids.

Univariate analysis of the plasma lipidome identified a subset of lipid species that differed significantly between patients before AI therapy (BRC group) and those receiving AI therapy (AI group) (Figure 3). Although no individual lipid species remained significant after Benjamini–Hochberg false discovery rate correction, the lipid alterations showed systematic class-level trend which was significant for several lipid classes under Fisher’s combined probability test (as seen in Figure 1), and therefore, species-level results are still reported as nominally significant (unadjusted *p* < 0.05). The volcano plot depicts the log_2_ fold change (AI vs. BRC) plotted against the −log_10_-transformed *p*-values obtained from the Mann–Whitney U test. Lipid species exceeding the predefined significance threshold are highlighted.

Several lipid species showed significantly higher levels in the BRC group (negative log_2_ fold change). These included multiple phosphatidylcholines (PC 34:1, PC 34:2, PC 36:2, PC 36:4, PC 18:1_18:2), sphingomyelins (SM d34:1, SM d18:0/22:3, SM d18:2/24:1), and lysophosphatidylethanolamines (LPE 22:0, LPE 20:2, LPE 20:1, LPC 24:1, LPE 22:6). In contrast, lipid species significantly increased in the AI group (positive log_2_ fold change) comprised several ceramides (Cer d16:1/24:0, Cer d18:2/23:0, Cer 16:1/22:0), two hexosylceramides (HexCer d18:1/22:1, HexCer d16:1/16:1), phosphatidylinositol (PI 16:0_16:0), one diacylglycerol species (DG 16:0_20:4) and one triacylglycerol species (TG 50:2).

The majority of detected lipid species did not differ significantly between groups and clustered around a log_2_ fold change of zero. Overall, the univariate analysis revealed distinct differences in specific lipid species between the BRC and AI groups despite the absence of significant differences in conventional serum lipid parameters.

### 2.6. Classification Performance of Lipid Classes

**Figure 4:**
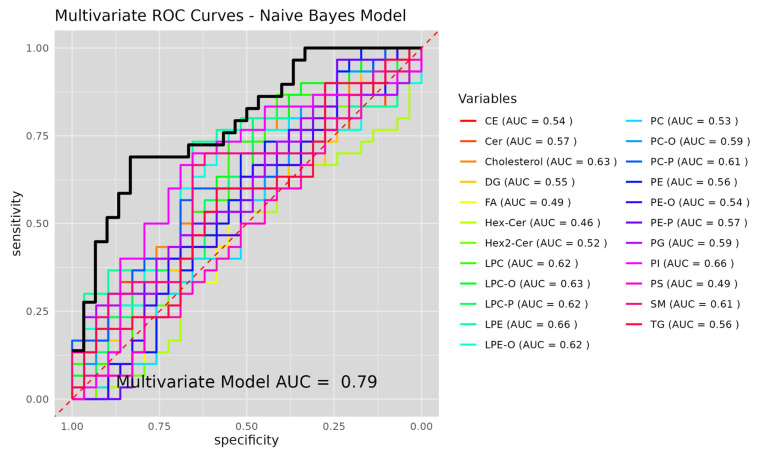
Receiver operating characteristic (ROC) curves showing the classification performance of individual lipid classes and a combined multivariate model in distinguishing breast cancer patients before aromatase inhibitor therapy (BRC) from patients receiving aromatase inhibitors (AI). ROC curves were generated using a Naive Bayes classifier. The area under the curve (AUC) is indicated for each lipid class, and the dashed diagonal line represents random classification. The combined multivariate model achieved an AUC of 0.79 (black line).

Receiver operating characteristic (ROC) curve analysis was performed to evaluate the discriminatory performance of individual lipid classes and a combined multivariate model in distinguishing between the BRC and AI groups (Figure 4). ROC curves were generated using a Naive Bayes classification approach, and the area under the curve (AUC) was calculated for each lipid class. Among individual lipid classes, the AUC values ranged from 0.46 to 0.66. The highest AUC values were observed for phosphatidylinositols (PI, AUC = 0.66), lysophosphatidylethanolamines (LPE, AUC = 0.66), cholesterol (AUC = 0.63), ether- and plasmalogen lysophosphatidylcholines (LPC-O, AUC = 0.63; LPC-P, AUC = 0.62), and lysophosphatidylcholines (LPC, AUC = 0.62). Moderate classification performance was also observed for sphingomyelins (SM, AUC = 0.61), plasmalogen phosphatidylcholines (PC-P, AUC = 0.61), ether phosphatidylcholines (PC-O, AUC = 0.59), phosphatidylglycerols (PG, AUC = 0.59), and ceramides (Cer, AUC = 0.57). Lower AUC values were obtained for fatty acids (FA, AUC = 0.49), phosphatidylserines (PS, AUC = 0.49), monohexosylceramides (Hex-Cer, AUC = 0.46), cholesteryl esters (CE, AUC = 0.54), diacylglycerols (DG, AUC = 0.55), phosphatidylethanolamines (PE, AUC = 0.56), ether phosphatidylethanolamines (PE-O, AUC = 0.54), plasmalogen phosphatidylethanolamines (PE-P, AUC = 0.57), triacylglycerols (TG, AUC = 0.56), and dihexosylceramides (Hex2-Cer, AUC = 0.52). The combined multivariate Naive Bayes model incorporating lipid class-level information demonstrated improved discrimination between the BRC and AI groups, achieving an overall AUC of 0.79. An independent Logistic Regression model was also calculated, which achieved AUC of 0.94 (Supplementary Figure S2). Classification performance metrics of the Naive Bayes and Logistic Regression models are presented for exploratory purposes, and the AUC values should be interpreted with caution given the lack of explicit cross-validation or external validation.

### 2.7. Hierarchical Clustering of Lipid Classes

**Figure 5:**
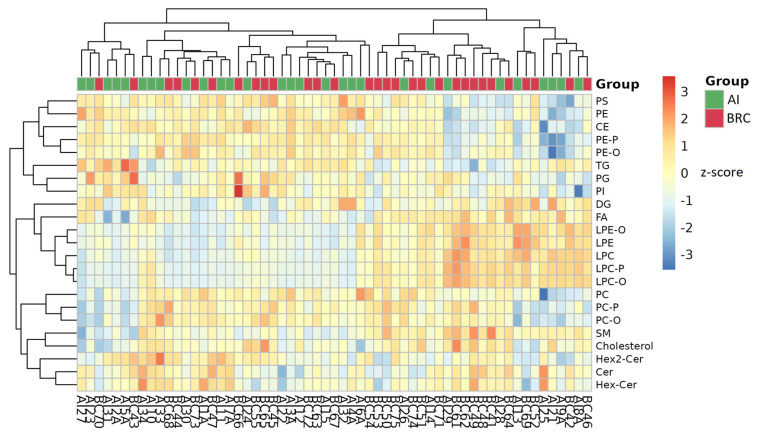
Heatmap with hierarchical clustering of lipid class-level plasma profiles in breast cancer patients before aromatase inhibitor therapy (BRC) and patients receiving aromatase inhibitors (AI). Rows represent lipid classes and columns represent individual samples. Data are shown as z-score-scaled lipid class intensities. Samples and lipid classes were clustered using unsupervised hierarchical clustering. Group color labels are indicated by the color bar above the heatmap as green (AI) and red (BRC).

Hierarchical clustering analysis was performed on lipid class-level data to assess global patterns of lipid abundance across individual samples from the BRC and AI groups (Figure 5). The heatmap displays scaled lipid class intensities (z-scores) for each sample, with rows representing lipid classes and columns representing individual patients. Samples are annotated according to group membership (AI or BRC), and both samples and lipid classes were clustered using unsupervised hierarchical clustering. The clustering revealed heterogeneity in lipid class abundance across samples, with partial grouping of patients according to AI therapy status. Several lipid classes, including glycerophospholipids, sphingolipids, neutral lipids, and lysophospholipids, exhibited variable abundance patterns across the cohort. Ether-linked and plasmalogen lipid subclasses were included alongside their corresponding diacyl counterparts, contributing to the overall clustering structure. At the lipid class level, differences in relative abundance were observed across samples for multiple classes, including phosphatidylcholines (PC, PC-O, PC-P), phosphatidylethanolamines (PE, PE-O, PE-P), lysophospholipids (LPC, LPC-O, LPC-P, LPE, LPE-O), sphingolipids (SM, Cer, HexCer, Hex2Cer), neutral lipids (TG, DG, CE), phosphatidylinositols (PI), phosphatidylglycerols (PG), phosphatidylserines (PS), fatty acids (FA), and cholesterol. The dendrograms indicate similarity relationships among lipid classes as well as among samples based on their lipidomic profiles.

### 2.8. Network Analysis of Lipid-Related Enzymes and Pathways

**Figure 6:**
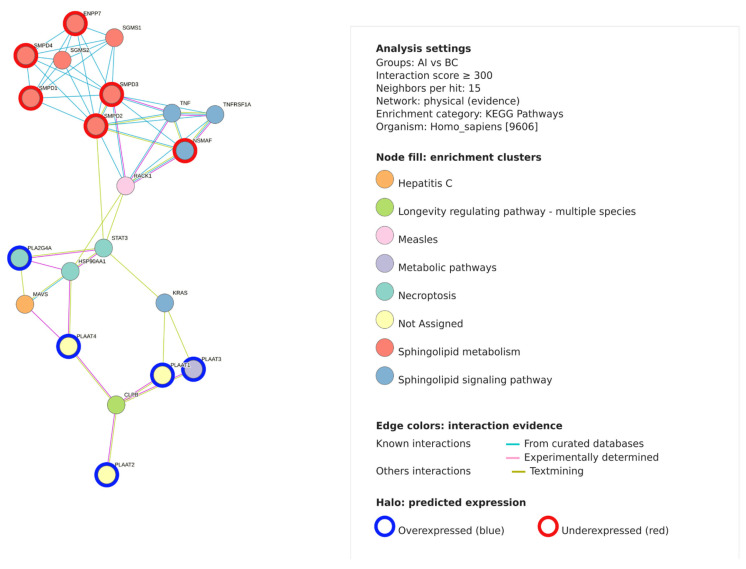
Protein–protein interaction network of lipid-associated enzymes and signaling proteins linked to differential lipidomic profiles between breast cancer patients before aromatase inhibitor therapy (BRC) and patients receiving aromatase inhibitors (AI). The network was generated using experimentally supported physical interactions (interaction score ≥ 300) and KEGG pathway enrichment analysis. Node colors indicate enriched KEGG pathways, and edge colors represent interaction evidence derived from curated databases, experimental data, or text-mining sources. Node halos denote predicted expression status, with blue indicating overexpression and red indicating underexpression. This network analysis is inferred solely from lipidomic data and does not represent experimentally measured gene or protein expression changes.

A protein–protein interaction network (based on the LipidOne 2.4) was constructed to explore associations between lipid-related enzymes linked to the differential lipid profiles observed between the AI and BRC groups (Figure 6). The network was generated using experimentally supported physical interactions with a minimum interaction score of 300 and up to 15 neighboring nodes per protein. Pathway enrichment analysis was performed using KEGG pathway annotations for Homo sapiens. The resulting network comprised several interconnected clusters, with nodes representing proteins and edges indicating known or predicted interactions. Nodes were grouped according to enriched KEGG pathways. Edge colors denote interaction evidence derived from curated databases, experimentally determined interactions, or text-mining sources. Within the network, multiple enzymes involved in sphingolipid metabolism and signaling were present, including sphingomyelinases (SMPD1, SMPD2, SMPD3, SMPD4), sphingomyelin synthases (SGMS1, SGMS2), and ectonucleotide pyrophosphatase/phosphodiesterase 7 (ENPP7). Additional nodes included phospholipase A family members (PLAAT1–PLAAT4, PLA2G4A), signaling-related proteins (TNF, TNFRSF1A, NSMAF, STAT3, KRAS), and molecular chaperones (HSP90AA1). Node halos indicate the directionality of lipid-associated changes mapped to lipid-related enzymes, representing putative pathway involvement based on lipidomic data. As this analysis is inferred solely from lipidomic profiles, it should be interpreted as hypothesis-generating and does not directly reflect gene or protein expression changes.

## 3. Discussion

Although AIs substantially improve long-term survival and reduce mortality in BRC patients, the profound reduction in estrogen levels induced by these therapies has important metabolic consequences. Estrogen plays a central role in regulating lipid homeostasis at both central and peripheral levels; therefore, its depletion may contribute to therapy-associated metabolic alterations. Estrogen promotes lipolysis by enhancing hepatic fatty acid (FA) oxidation and suppresses lipogenesis, thereby limiting hepatic lipid accumulation [15,16]. At the molecular level, estrogen inhibits key lipogenic pathways by suppressing fatty acid synthase (FASN) activity and downregulating stearoyl-CoA desaturase 1 (SCD-1), a critical enzyme in monounsaturated fatty acid (MUFA) synthesis [17]. In addition, estrogen receptor (ER) signaling negatively regulates cholesterol biosynthesis through the inhibition of HMG-CoA reductase and reduced activation of sterol regulatory element-binding protein 2 (SREBF2) [18,19]. Collectively, these mechanisms provide a biological framework for understanding how estrogen deprivation induced by AI therapy may lead to alterations in lipid metabolism. Such changes are likely to be reflected in the circulating lipidome, underscoring the relevance of lipidomic profiling as a sensitive approach to capture AI-associated metabolic perturbations in postmenopausal BRC patients.

Using targeted lipidomics profiling, we quantified a broad spectrum of lipid classes, including glycerophospholipids, sphingolipids, glycerolipids, free fatty acids, cholesteryl esters, and cholesterol. Univariate analysis revealed a limited number of lipid species that differed significantly between patients before initiation of the AI therapy and those receiving AI treatment. Lipid species most significantly increased in AI-treated patients included ceramides Cer d18:1/24:0, Cer d18:2/23:0, and Cer d16:1/22:0, a hexosylceramides (HexCer d18:1/22:1 and HexCer d16:1/16:1), phosphatidylinositol PI 16:0_16:0, and a polyunsaturated triacylglycerol species TG 50:2 (FA 18:2). In contrast, lipid species that were most significantly abundant in the BRC group comprised several phosphatidylcholines (PC 34:1, PC 34:2, PC 36:2, PC 36:4, PC 18:1_18:2), sphingomyelins (SM d34:1, SM d18:0/22:3, SM d18:2/24:1), and lysophosphatidylethanolamines (LPE 22:0, LPE 24:1, and LPE 22:6).

Sphingolipids are essential structural components of cellular membranes and exert diverse biological functions, including regulation of apoptosis, cell proliferation, inflammation, nutrient uptake, stress responses, and autophagy [20]. Their relevance is particularly pronounced in the context of cancer therapy, as cancer cell lines deficient in ceramide synthesis often exhibit resistance to chemotherapy and radiotherapy [21]. Additionally, ceramides’ fatty acyl chain length may be a key factor influencing their molecular actions. Importantly, the biological activity of ceramides is influenced by the length and saturation of their fatty acyl chains. For example, Kim et al. demonstrated that the selective elevation of medium-chain saturated ceramides (C14:0, C16:0, and C18:0) in MCF-7 breast cancer cells suppressed cell proliferation by inhibiting mTOR signalling [22]. In line with this, decreased levels of long-chain ceramides with saturated FA (C20:0, C22:0, C24:0, and C26:0) have been associated with increased metastatic potential and poorer prognosis in BRC patients [23], suggesting that elevation of both medium- and long-chain Cer with saturated FA is generally correlated with favorable outcomes in these patients. In this study, we observed an increase in certain ceramides, such as Cer d16:1/24:0, Cer d18:2/23:0, and Cer 16:1/22:0, which contain saturated C22:0, C23:0 and C24:0. These alterations may reflect antitumor-related lipid remodeling associated with AI therapy, potentially involving ceramide accumulation. Specifically, a negative correlation of estrogen with serum ceramide (specifically d18:1/24:1) levels had been found in postmenopausal women [24]. Furthermore, estradiol significantly reduces levels of ceramides d18:1/16:0, d18:1/24:0, and d18:1/24:1 in the MCF 7 cell line, indicating that estradiol may downregulate the biosynthesis or upregulate the degradation of these lipids [24]. More precisely, 17β-estradiol upregulates expression of specific ceramide synthase isoforms, including CerS4 and CerS5, through estrogen receptor-mediated activation of transcription factors such as AP-1, and directly increases ceramide production by enhancing enzyme expression at the transcriptional level [25]. Aromatase inhibitors reduce circulating estrogen levels by directly blocking the activity of the aromatase enzyme (CYP19A1), by inhibiting its active site, which catalyzes the conversion of androstenedione and testosterone into estrone and estradiol, thereby suppressing estrogen biosynthesis, particularly in postmenopausal women, where peripheral aromatisation is the primary source of estrogen [26]. Since AIs decrease systemic estrogen levels, the observed increase in certain ceramides in the AI group in our study may reflect the effects of estrogen deprivation.

Although significant accumulation of certain Cer in plasma, muscle, brain and liver, including (d18:1/18:0), (d18:1/22:0), (d18:1/24:0), and (d18:1/24:1) can be linked with several conditions such as atherosclerosis [27], obesity [28], hypertension [29], and other cardiovascular diseases [30]. We did not observe significant changes in these ceramide species in our patients. However, in our study, ceramides containing other MUFA and SFA species were also elevated, and to the best of our knowledge, no previous studies have linked these specific ceramide species to metabolic alterations. Therefore, further research is warranted to elucidate the metabolic consequences associated with these ceramide species.

Once generated, ceramides can be directly metabolized by ceramide kinase (Cerk), acid ceramidase 1, or sphingomyelin synthase 1/2 to produce ceramide 1-phosphate, sphingosine, or sphingomyelin (SM), respectively [31]. SM promotes cell proliferation, and an increase in plasma SM (d18:1/24:0) and SM (d18:1/24:1) levels was detected in women with BRC compared with healthy women [32]. Our study identified significantly reduced levels of these SM species in the AI group, suggesting antiproliferative effects of AI mediated through decreased SM levels. This effect may be driven by inhibition of ceramidase activity, resulting in increased ceramide levels and decreased SM synthesis, a mechanism that has previously been reported for tamoxifen [33,34,35]. Additionally, Cer is converted into hexosylceramides (HexCer), a class of ceramide metabolites characterized by the presence of a neutral sugar moiety attached to the ceramide backbone [36]. HexCer is known to play a significant role in resistance to breast cancer therapies, with low levels of HexCer correlating with chemotherapy resistance in BRC cell lines [37]. In our study, specifically, the HexCer d18:1/22:1 was found to be significantly increased in the patients receiving AI therapy.

Further, phosphatidylcholines (PC, such as PC 36:4, PC 34:1, PC 34:2, and PC 18:1_18:2), lysophosphatidylcholines (LPC, such as LPC 24:1 and LPC 20:5), and lysophosphatidylethanolamines (LPE, such as LPE 20:1, LPE 24:1, LPE 22:0, and LPE 22:6) were increased in the plasma of BRC patients before AI therapy whereas phosphatidylinositols (PI, such as PI 32:0, specifically PI 16:0_16:0) were increased in patients receiving AI therapy. PC is the most prevalent phospholipid in cellular membranes and plays a crucial structural role in cell membranes and lipoproteins [38]. Degradation of PC yields two important secondary messengers, phosphatidic acid and choline, which are involved in various signaling pathways [32]. Other metabolites derived from PC, such as LPC, can stimulate cell proliferation and survival through G protein-coupled receptors. There is evidence that the level of some PC is decreased in cancer tissue [39] or serum of BRC patients [32]. Specifically, lower levels of PC (18:1/22:2) and PC (18:0/18:0) and higher levels of LPC (16:4) have been reported in the serum of BRC patients compared to healthy controls [32]. The relative reduction in the PC and LPC species following AI therapy shown in our data suggests therapy-associated modulation of phosphatidylcholine turnover and lysophospholipid availability, contributing to distinct plasma lipidomic profiles before and after endocrine treatment. In addition, circulating lipidomics in lung cancer has been reported to show decreased LPE/PE levels in patients relative to controls, further supporting that reduced LPE can be detected in blood-based lipidomes in certain malignancies [40]. At the cellular level, lipidomic profiling of triple-negative breast cancer cells identified lower LPE abundance in specific phenotypic subsets, indicating that LPE differences may also be observed in breast cancer-derived models, although these data are not directly comparable to plasma measurements [41]. Taken together, prior studies suggest that decreases in circulating LPE are plausible in cancer-related lipidomic phenotypes; however, the biological basis and clinical implications of reduced plasma LPE, particularly in the context of endocrine therapy, remain to be clarified and warrant further investigation.

PI, although found in low amounts in lipoprotein particles, play central roles in regulating cell growth, survival, intracellular trafficking, and cytoskeletal organization in breast cancer cells, primarily through activation of the PI3K/AKT/mTOR signalling pathway [42]. Merchant et al. (1991) reported elevated concentrations of PI in breast cancer tissue, and a specific PI species (22:0/22:4) was found at higher levels in malignant serum samples [32,43]. Conversely, other studies have demonstrated decreased levels of PI 16:0/16:1 and PI 18:0/20:4 in the plasma of BRC patients, suggesting these molecules may serve as potential biomarkers for breast cancer [44]. Previously we have also found that two abundant PI species containing palmitic (16:0) and stearic (18:0) acids, PI 16:0/18:1 and PI 18:0/18:1, were significantly downregulated in non-Hodgkin lymphoma patients, likely due to increased utilization in the PI3K signaling pathway [45]. In our study, AI therapy led to an increase in only one PI molecule: PI 16:0_16:0. Given the critical role of phosphatidylinositol 3,4,5-triphosphate (PIP3), as PI metabolite, in cancer development—regulating cell growth, survival, and migration [46], and the fact that some PI species are elevated while others are decreased in the plasma of BRC patients, further studies are needed to better understand PI’s role in breast cancer progression and treatment. Also, there is currently no direct evidence supporting a specific biological function for the saturated PI 16:0_16:0 species in breast cancer, and its potential contribution to disease-related signalling; therefore, this warrants further investigation.

Elevated TG levels, key constituents of very low-density lipoproteins (VLDL), are closely associated with the development of atherosclerosis and hyperlipoproteinemia, thereby increasing the risk of cardiovascular complications. Previous studies have reported that saturated TG species, such as TG (50:0) and TG (54:0), are elevated in the plasma of individuals with breast cancer, implicating these molecules in tumor progression [44]. In our study, one TG species, TG 50:2 (containing FA18:2), was increased in the AI group.

Although AI therapy has frequently been associated with an increased risk of cardiovascular complications, our study did not demonstrate significant alterations in lipid classes that are well-established contributors to cardiovascular disease. Specifically, lipid species within the ceramide, phospholipid, and triglyceride classes that are typically elevated in metabolic and cardiovascular conditions such as obesity, insulin resistance, atherosclerosis, and hypertension remained largely unchanged in our patient cohort. While certain lipid species were increased in patients receiving AI therapy, the clinical relevance of these changes with respect to cardiovascular risk remains unclear. Given the limited sample size of our study, further investigations in larger cohorts are required to more comprehensively assess the potential cardiometabolic effects of AI therapy. Nevertheless, our results suggest a potentially protective, antitumor-related effect of AI therapy, as evidenced by favorable alterations in specific lipid molecules that may be associated with reduced tumor cell proliferation and a lower risk of disease recurrence and metastatic progression.

This study has several limitations. Given the cross-sectional design and use of independent cohorts, observed lipidomic differences should be interpreted as associations rather than solely therapy-induced effects. Furthermore, future longitudinal studies with paired pre- and post-treatment samples from the same individuals, enabling within-subject baseline correction, are needed to more accurately determine therapy-related lipidomic changes. The relatively small sample size may limit statistical power and generalizability and should be considered as an exploratory study. In addition, plasma lipid profiles may not fully reflect tissue-specific lipid metabolism, and residual confounding by BMI (ranging from 19.2 to 38 kg/m^2^) and lifestyle factors cannot be excluded. Furthermore, although all participants were treated according to the same therapeutic protocols, the BRC group had a disease duration and treatment period of approximately one year, whereas the AI group had 3–4 years since diagnosis and treatment initiation, which may influence lipid profiles. Finally, the findings are limited to patients receiving non-steroidal AIs and may not apply to other endocrine treatment regimens.

This study also has several strengths. All analyses were performed in the same laboratory using standardized protocols, minimizing analytical variability. Plasma samples were collected from two different hospitals, enhancing representativeness. Strict inclusion criteria excluded patients on statins or with metabolic comorbidities, while participants were not taking supplements and maintained a relatively uniform diet, reducing potential confounding. Medication use was limited to AIs and, in some cases, antihypertensives, further limiting variability. Moreover, all patients underwent surgery and completed the same adjuvant chemotherapy protocols. These features increase the reliability of the observed associations between AI therapy and plasma lipidomic profiles.

## 4. Materials and Methods

### 4.1. Patients

This study included 29 postmenopausal women with a history of breast cancer who had been receiving AI therapy for at least two years (AI group) and 30 postmenopausal women with breast cancer who had undergone surgery but had not yet initiated AI treatment (BRC group). Participants were recruited from the University Hospital Medical Center “Bežanijska kosa,” Belgrade, Serbia, and the Military Medical Academy, Belgrade, Serbia, between 2020 and 2022. The diagnosis of breast cancer was histologically confirmed in all patients. Estrogen receptor (ER) expression was determined by immunohistochemical analysis performed at the hospital.

Eligible participants were postmenopausal women with histopathologically confirmed stage I–IIIA breast cancer characterized by ER-positive and/or PR-positive, HER2-negative tumors, who had undergone complete surgical resection, and followed by chemotherapy according to the AC-T protocol prior to recruitment. Exclusion criteria included metastatic disease, HER2-positive breast cancer, a history of stroke or myocardial infarction, diabetes, autoimmune diseases, significant neurological deficits or disorders of consciousness, dementia, other malignancies, and the use of statins or dietary supplements known to affect lipid metabolism.

Adjuvant endocrine therapy consisted of non-steroidal aromatase inhibitors, specifically anastrozole (1 mg/day) or letrozole (2.5 mg/day), administered at a stable dose for a minimum duration of 24 months.

The study was approved by the Ethics Committee of the Military Medical Academy (No. 14/2020 on 8 February 2019) and the Medical Ethics Committee of the University Hospital Medical Center Bezanijska kosa, Faculty of Medicine, University of Belgrade (No. 6041/1 on 21 September 2022), adhering to the Declaration of Helsinki and Good Clinical Practice principles. All participants provided written informed consent before inclusion in the study.

### 4.2. Blood Collection and Anthropometric Measurements

Anthropometric measurements, including body weight, height and body mass index (BMI) were gathered using standardized procedures and calculations. Data on dietary intake were collected using three non-consecutive 24 h recalls including one weekend day, by a trained nutritionist. Blood samples for all biochemical analyses were collected in the morning after an overnight fast. Fasting glucose levels and serum lipid parameters—triglycerides (TG), total cholesterol (TC), high-density lipoprotein cholesterol (HDL), and low-density lipoprotein cholesterol (LDL)—were measured using an automated clinical chemistry analyzer Dimension RxL Max (Siemens Healthineers, Erlangen, Germany) and commercially available Roche diagnostic kits (Roche Diagnostics GmbH, Mannheim, Germany), in accordance with the manufacturer’s instructions.

Blood samples collected in EDTA-containing tubes were immediately placed on ice and centrifuged at 4000× *g* for 10 min at 4 °C to separate plasma. The resulting plasma samples were stored at −80 °C until submission to liquid chromatography–mass spectrometry (LC/MS) analysis.

### 4.3. Chemicals and Reagents

Acetonitrile (ACN), isopropanol (IPA), water, and ammonium acetate (all LC–MS grade) were obtained from Sigma-Aldrich (St. Louis, MO, USA). The SPLASH^®^ LIPIDOMIX^®^ Mass Spec Standard mixture, along with ceramide (d18:1-d7/15:0) and deuterated oleic acid (FA 18:1-d9), was purchased from Avanti Polar Lipids (Alabaster, AL, USA).

### 4.4. Lipid Extraction

To a sample of 50 µL of EDTA plasma, 150 µL of isopropanol containing the internal standard mixture (SPLASH^®^ LIPIDOMIX^®^ 3%, Cer d18:1-d7/15:0, and FA18:1 d9) was added as described previously [47]. Briefly, the mixture was vortexed and stored overnight at −80 °C to facilitate deproteinization. The deproteinized samples were centrifuged at 14,000× *g* for 20 min at 4 °C to separate the supernatant. A 100 µL of the supernatant was then transferred to a glass vial for LC-MS analysis, with an injection volume of 2 µL.

Quality control (QC) samples were prepared by combining 5 µL of supernatant from each experimental sample. These QC samples were used to normalize the LC-MS system drift and to assess its reproducibility and stability.

### 4.5. Lipidomic Analysis

Chromatographic separation was performed using an ultra-high-performance liquid chromatography (UHPLC) ExionLC system (SCIEX, Framingham, MA, USA) coupled to a QTRAP^®^ 6500+ mass spectrometer (SCIEX, Framingham, MA, USA). Lipids were separated on an ACQUITY Premier BEH C8 column (100 × 2.1 mm, 1.7 μm; Waters Corporation, Wilmslow, UK). The column temperature was kept at 55 °C, and the autosampler was set to 10 °C. The mobile phases consisted of solvent A (acetonitrile/water, 6:4, *v*/*v*, containing 10 mM ammonium acetate) and solvent B (isopropanol/acetonitrile, 9:1, *v*/*v*, containing 10 mM ammonium acetate), delivered at a flow rate of 0.4 mL/min. The total analytical run time was 20 min per sample. Data acquisition was carried out using Analyst software (version 1.6.2), and data processing and semiautomatic peak integration were performed with MultiQuant software (version 3.0; SCIEX, Framingham, MA, USA). Samples were analyzed in a randomized sequence with continuous monitoring of quality control samples. Raw peak areas were QC-normalized by LOESS [48] and IS-normalized by the corresponding IS class for each lipid.

### 4.6. Statistical Analysis

The data were processed using the Metabol package [48] in R program (v 3.6.3). Preprocessing of the data included locally estimated scatterplot smoothing (LOESS), Pareto scaling, filtering of analytes (coefficients of variation in the QC above 30%), mean centering, and the lnPQN normalization (Supplementary Table S1) [49]. Univariate statistical analysis (based on the non-parametric non-paired Mann–Whitney U test (*p*-values shown as −log) together with the log2 fold-change in medians) was performed and visualized using GraphPad Prism (version 9.0, GraphPad Software, LLC, Boston, MA, USA). Data were also evaluated using multivariate statistical analysis, namely the principal component analysis (PCA) and orthogonal discriminant analysis by partial least squares (OPLS-DA) and validated by permutation using SIMCA software (version 18.0, Sartorius Umetrics, Umeå, Sweden). Benjamini–Hochberg correction was used for false discovery rate correction. Detected lipid species were mapped to standardized lipid classes and subclasses according to accepted lipidomics nomenclature, enabling consistent grouping of lipid species for downstream statistical, class-level, and network analyses. LipidOne 2.4 was further used to support lipid-centric data integration and visualization prior to multivariate and pathway-based analyses (such as heatmaps, ROC analyses using a Naive-Bayesian model and Logistic Regression model, and protein–protein interaction network based on lipidomics data) [50]. Lipid network visualization was performed using Cytoscape software (v 3.8.2) [51]. Differentially abundant lipid species were organized according to lipid class and structural similarity. Node size reflected the −log10 *p*-value from univariate analysis, while node color represented log2 fold change between groups. Calculation of Fisher’s combined probability test for assessing systematic lipid class-level differences was performed (Supplementary Table S2) and calculated following procedures described previously [52].

## 5. Conclusions

In conclusion, our findings demonstrate significant alterations in lipid metabolism in BRC patients undergoing AI therapy, with pronounced changes in Cer, SM, DG, TG, PC, PI, and PE lipids and lipid classes. However, the precise mechanisms underlying the differences in lipid metabolism between patients before and during AI treatment remain to be fully elucidated. Future longitudinal studies with paired pre- and post-treatment samples from the same individuals leveraging comprehensive lipidomic profiling are needed to deepen our understanding of AI-induced metabolic alterations and to inform the development of targeted interventions aimed at mitigating adverse effects, ultimately improving long-term outcomes and quality of life for these patients.

## Figures and Tables

**Table 1:** Baseline anthropometric and clinic characteristics of included patients.

Variables	BRC Group (*n* = 30)	AI group (*n* = 29)	Mann–Whitney U Test (*p*-Value)
Age (years)	58 (15)	61 (12)	0.1379
Weight (kg)	76.0 (15.9)	72.8 (20.0)	0.6041
Height (cm)	162.9 (9.5)	163.0 (6.0)	0.9235
Body mass index (BMI (kg/m^2^))	29.89 (10.73)	27.56 (6.70)	0.7666
Breast cancer stage			
I	0	1	
IIa	13	13	
IIb	12	11	
IIIa	5	4	
Breast cancer subtype (case/total)			
Infiltrating ductal	66.66% (20/30)	68.96% (20/29)	
Infiltrating lobular	16.66% (5/30)	20.68% (6/29)	
Other	16.66% (5/30)	10.34% (3/29)	

For continuous variables, the data are presented as mean value (interquartile range).

**Table 2:** Biochemical characteristics of included patients.

Biochemical Parameter	BRC Group (*n* = 30)	AI Group (*n* = 29)	Mann–Whitney U Test (*p*-Value)
GLC (mmol/L)	5.9 (1.2)	6.1 (0.8)	0.4416
TG (mmol/L)	1.48 (0.82)	1.53 (0.77)	0.8080
CHOL (mmol/L)	5.57 (1.78)	5.82 (0.81)	0.3677
HDL-CHOL (mmol/L)	1.45 (0.40)	1.68 (0.55)	0.0221
LDL-CHOL (mmol/L)	3.47 (1.45)	3.48 (1.06)	0.9590

The values are presented as means (interquartile range). GLC—glucose, TG—triglycerides, CHOL—cholesterol, HDL-CHOL—High-density cholesterol, LDL-CHOL—Low-density cholesterol.

## Data Availability

The data presented in this study are available from the corresponding author upon request.

## Supplementary Materials

The following supporting information can be downloaded at: https://www.mdpi.com/article/10.3390/ijms27041926/s1.

## References

[1] Bray F., Laversanne M., Sung H., Ferlay J., Siegel R.L., Soerjomataram I., Jemal A. (2024). Global Cancer Statistics 2022: GLOBOCAN Estimates of Incidence and Mortality Worldwide for 36 Cancers in 185 Countries. CA Cancer J. Clin..

[2] Sleightholm R., Neilsen B.K., Elkhatib S., Flores L., Dukkipati S., Zhao R., Choudhury S., Gardner B., Carmichael J., Smith L. (2021). Percentage of Hormone Receptor Positivity in Breast Cancer Provides Prognostic Value: A Single-Institute Study. J. Clin. Med. Res..

[3] Yu K.-D., Wu J., Shen Z.-Z., Shao Z.-M. (2012). Hazard of Breast Cancer-Specific Mortality among Women with Estrogen Receptor-Positive Breast Cancer after Five Years from Diagnosis: Implication for Extended Endocrine Therapy. J. Clin. Endocrinol. Metab..

[4] Goss P.E., Ingle J.N., Pritchard K.I., Robert N.J., Muss H., Gralow J., Gelmon K., Whelan T., Strasser-Weippl K., Rubin S. (2016). Extending Aromatase-Inhibitor Adjuvant Therapy to 10 Years. N. Engl. J. Med..

[5] Jeong S., Woo M.M., Flockhart D.A., Desta Z. (2009). Inhibition of Drug Metabolizing Cytochrome P450s by the Aromatase Inhibitor Drug Letrozole and Its Major Oxidative Metabolite 4,4′-Methanol-Bisbenzonitrile in Vitro. Cancer Chemother. Pharmacol..

[6] Bérczi B., Farkas N., Hegyi P., Tóth B., Csupor D., Németh B., Lukács A., Czumbel L.M., Kerémi B., Kiss I. (2024). Aromatase Inhibitors and Plasma Lipid Changes in Postmenopausal Women with Breast Cancer: A Systematic Review and Meta-Analysis. J. Clin. Med..

[7] De Buttros D.A.B., Branco M.T., Orsatti C.L., de Almeida-Filho B.S., Nahas-Neto J., Nahas E.A.P. (2019). High Risk for Cardiovascular Disease in Postmenopausal Breast Cancer Survivors. Menopause.

[8] Kvasnička A., Najdekr L., Dobešová D., Piskláková B., Ivanovová E., Friedecký D. (2023). Clinical Lipidomics in the Era of the Big Data. Clin. Chem. Lab. Med..

[9] Cífková E., Brumarová R., Ovčačíková M., Dobešová D., Mičová K., Kvasnička A., Vaňková Z., Šiller J., Sákra L., Friedecký D. (2022). Lipidomic and Metabolomic Analysis Reveals Changes in Biochemical Pathways for Non-Small Cell Lung Cancer Tissues. Biochim. Biophys. Acta Mol. Cell Biol. Lipids.

[10] Wolrab D., Jirásko R., Cífková E., Höring M., Mei D., Chocholoušková M., Peterka O., Idkowiak J., Hrnčiarová T., Kuchař L. (2022). Lipidomic Profiling of Human Serum Enables Detection of Pancreatic Cancer. Nat. Commun..

[11] Jirásko R., Idkowiak J., Wolrab D., Kvasnička A., Friedecký D., Polański K., Študentová H., Študent V., Melichar B., Holčapek M. (2022). Altered Plasma, Urine, and Tissue Profiles of Sulfatides and Sphingomyelins in Patients with Renal Cell Carcinoma. Cancers.

[12] Santiago P., Melo T., Barceló-Nicolau M., Barceló-Coblijn G., Domingues P., Domingues R. (2025). Advancing Colorectal Cancer Research through Lipidomics. Mol. Omics.

[13] Carmona A., Mitri S., James T.A., Ubellacker J.M. (2024). Lipidomics and Metabolomics as Potential Biomarkers for Breast Cancer Progression. npj Metab. Health Dis..

[14] Pan M., Qin C., Han X. (2021). Lipid Metabolism and Lipidomics Applications in Cancer Research. Adv. Exp. Med. Biol..

[15] Mahboobifard F., Pourgholami M.H., Jorjani M., Dargahi L., Amiri M., Sadeghi S., Tehrani F.R. (2022). Estrogen as a Key Regulator of Energy Homeostasis and Metabolic Health. Biomed. Pharmacother..

[16] Palmisano B.T., Zhu L., Stafford J.M. (2017). Role of Estrogens in the Regulation of Liver Lipid Metabolism. Adv. Exp. Med. Biol..

[17] Bryzgalova G., Lundholm L., Portwood N., Gustafsson J.-A., Khan A., Efendic S., Dahlman-Wright K. (2008). Mechanisms of Antidiabetogenic and Body Weight-Lowering Effects of Estrogen in High-Fat Diet-Fed Mice. Am. J. Physiol. Endocrinol. Metab..

[18] Pedram A., Razandi M., O’Mahony F., Harvey H., Harvey B.J., Levin E.R. (2013). Estrogen Reduces Lipid Content in the Liver Exclusively from Membrane Receptor Signaling. Sci. Signal..

[19] Li Y., Deng Z., Wang Y., Shen S. (2024). Lipid Changes during Endocrine Therapy in Early-Stage Breast Cancer Patients: A Real-World Study. Lipids Health Dis..

[20] Borodzicz-Jażdżyk S., Jażdżyk P., Łysik W., Cudnoch-Jȩdrzejewska A., Czarzasta K. (2022). Sphingolipid Metabolism and Signaling in Cardiovascular Diseases. Front Cardiovasc. Med..

[21] Pal P., Atilla-Gokcumen G.E., Frasor J. (2022). Emerging Roles of Ceramides in Breast Cancer Biology and Therapy. Int. J. Mol. Sci..

[22] Kim M.H., Park J.-W., Lee E.-J., Kim S., Shin S.-H., Ahn J.-H., Jung Y., Park I., Park W.-J. (2018). C16-ceramide and Sphingosine 1-phosphate/S1PR2 Have Opposite Effects on Cell Growth through mTOR Signaling Pathway Regulation. Oncol. Rep..

[23] Pani T., Rajput K., Kar A., Sharma H., Basak R., Medatwal N., Saha S., Dev G., Kumar S., Gupta S. (2021). Alternative Splicing of Ceramide Synthase 2 Alters Levels of Specific Ceramides and Modulates Cancer Cell Proliferation and Migration in Luminal B Breast Cancer Subtype. Cell Death Dis..

[24] Vozella V., Basit A., Piras F., Realini N., Armirotti A., Bossù P., Assogna F., Sensi S.L., Spalletta G., Piomelli D. (2019). Elevated Plasma Ceramide Levels in Post-Menopausal Women: A Cross-Sectional Study. Aging.

[25] Wegner M.-S., Wanger R.A., Oertel S., Brachtendorf S., Hartmann D., Schiffmann S., Marschalek R., Schreiber Y., Ferreirós N., Geisslinger G. (2014). Ceramide Synthases CerS4 and CerS5 Are Upregulated by 17β-Estradiol and GPER1 via AP-1 in Human Breast Cancer Cells. Biochem. Pharmacol..

[26] Generali D., Berardi R., Caruso M., Cazzaniga M., Garrone O., Minchella I., Paris I., Pinto C., De Placido S. (2024). Corrigendum: Aromatase Inhibitors: The Journey from the State of the Art to Clinical Open Questions. Front. Oncol..

[27] Ichi I., Nakahara K., Miyashita Y., Hidaka A., Kutsukake S., Inoue K., Maruyama T., Miwa Y., Harada-Shiba M., Tsushima M. (2006). Association of Ceramides in Human Plasma with Risk Factors of Atherosclerosis. Lipids.

[28] Samad F., Hester K.D., Yang G., Hannun Y.A., Bielawski J. (2006). Altered Adipose and Plasma Sphingolipid Metabolism in Obesity: A Potential Mechanism for Cardiovascular and Metabolic Risk. Diabetes.

[29] Spijkers L.J.A., van den Akker R.F.P., Janssen B.J.A., Debets J.J., De Mey J.G.R., Stroes E.S.G., van den Born B.-J.H., Wijesinghe D.S., Chalfant C.E., MacAleese L. (2011). Hypertension Is Associated with Marked Alterations in Sphingolipid Biology: A Potential Role for Ceramide. PLoS ONE.

[30] de Mello V.D.F., Lankinen M., Schwab U., Kolehmainen M., Lehto S., Seppänen-Laakso T., Oresic M., Pulkkinen L., Uusitupa M., Erkkilä A.T. (2009). Link between Plasma Ceramides, Inflammation and Insulin Resistance: Association with Serum IL-6 Concentration in Patients with Coronary Heart Disease. Diabetologia.

[31] Arana L., Gangoiti P., Ouro A., Trueba M., Gómez-Muñoz A. (2010). Ceramide and Ceramide 1-Phosphate in Health and Disease. Lipids Health Dis..

[32] More T.H., Bagadi M., RoyChoudhury S., Dutta M., Uppal A., Mane A., Santra M.K., Chaudhury K., Rapole S. (2017). Comprehensive Quantitative Lipidomic Approach to Investigate Serum Phospholipid Alterations in Breast Cancer. Metabolomics.

[33] Zheng K., Chen Z., Feng H., Chen Y., Zhang C., Yu J., Luo Y., Zhao L., Jiang X., Shi F. (2019). Sphingomyelin Synthase 2 Promotes an Aggressive Breast Cancer Phenotype by Disrupting the Homoeostasis of Ceramide and Sphingomyelin. Cell Death Dis..

[34] Li R.-Z., Wang X.-R., Wang J., Xie C., Wang X.-X., Pan H.-D., Meng W.-Y., Liang T.-L., Li J.-X., Yan P.-Y. (2022). The Key Role of Sphingolipid Metabolism in Cancer: New Therapeutic Targets, Diagnostic and Prognostic Values, and Anti-Tumor Immunotherapy Resistance. Front. Oncol..

[35] Pal P., Millner A., Semina S.E., Huggins R.J., Running L., Aga D.S., Tonetti D.A., Schiff R., Greene G.L., Atilla-Gokcumen G.E. (2022). Endocrine Therapy-Resistant Breast Cancer Cells Are More Sensitive to Ceramide Kinase Inhibition and Elevated Ceramide Levels Than Therapy-Sensitive Breast Cancer Cells. Cancers.

[36] Kartal Yandım M., Apohan E., Baran Y. (2013). Therapeutic Potential of Targeting Ceramide/glucosylceramide Pathway in Cancer. Cancer Chemother. Pharmacol..

[37] Zhang X., Li J., Qiu Z., Gao P., Wu X., Zhou G. (2009). Co-Suppression of MDR1 (multidrug Resistance 1) and GCS (glucosylceramide Synthase) Restores Sensitivity to Multidrug Resistance Breast Cancer Cells by RNA Interference (RNAi). Cancer Biol. Ther..

[38] Li M., Fan P., Wang Y. (2015). Lipidomics in Health and Diseases—Beyond the Analysis of Lipids. J. Glycomics Lipidomics.

[39] Katz-Brull R., Lavin P.T., Lenkinski R.E. (2002). Clinical Utility of Proton Magnetic Resonance Spectroscopy in Characterizing Breast Lesions. J. Natl. Cancer Inst..

[40] Lv J., Gao D., Zhang Y., Wu D., Shen L., Wang X. (2018). Heterogeneity of Lipidomic Profiles among Lung Cancer Subtypes of Patients. J. Cell. Mol. Med..

[41] Huang X., Di X., Zuiderwijk M.C., Zhang L., Leegwater H., Davidse S., Kindt A., Harms A., Hankemeier T., Le Dévédec S.E. (2025). Lipidomic Profiling of Triple-Negative Breast Cancer Cells Reveals Distinct Metabolic Signatures Associated with EpCAM Expression. Talanta.

[42] Jin Y., Xue J. (2023). Lipid Kinases PIP5Ks and PIP4Ks: Potential Drug Targets for Breast Cancer. Front. Oncol..

[43] Merchant T.E., Meneses P., Gierke L.W., Den Otter W., Glonek T. (1991). 31P Magnetic Resonance Phospholipid Profiles of Neoplastic Human Breast Tissues. Br. J. Cancer.

[44] Yang L., Cui X., Zhang N., Li M., Bai Y., Han X., Shi Y., Liu H. (2015). Comprehensive Lipid Profiling of Plasma in Patients with Benign Breast Tumor and Breast Cancer Reveals Novel Biomarkers. Anal. Bioanal. Chem..

[45] Masnikosa R., Pirić D., Post J.M., Cvetković Z., Petrović S., Paunović M., Vučić V., Bindila L. (2023). Disturbed Plasma Lipidomic Profiles in Females with Diffuse Large B-Cell Lymphoma: A Pilot Study. Cancers.

[46] Wymann M.P., Schneiter R. (2008). Lipid Signalling in Disease. Nat. Rev. Mol. Cell Biol..

[47] Kvasnička A., Friedecký D., Brumarová R., Pavlíková M., Pavelcová K., Mašínová J., Hasíková L., Závada J., Pavelka K., Ješina P. (2023). Alterations in Lipidome Profiles Distinguish Early-Onset Hyperuricemia, Gout, and the Effect of Urate-Lowering Treatment. Arthritis Res. Ther..

[48] Gardlo A. (2019). AlzbetaG/Metabol.

[49] Dieterle F., Ross A., Götz Schlotterbeck A., Senn H. (2006). Probabilistic Quotient Normalization as Robust Method to Account for Dilution of Complex Biological Mixtures. Application in 1H NMR Metabonomics. Anal. Chem..

[50] Alabed H.B.R., Mancini D.F., Buratta S., Calzoni E., Giacomo D.D., Emiliani C., Martino S., Urbanelli L., Pellegrino R.M. (2024). LipidOne 2.0: A Web Tool for Discovering Biological Meanings Hidden in Lipidomic Data. Curr. Protoc..

[51] Shannon P., Markiel A., Ozier O., Baliga N.S., Wang J.T., Ramage D., Amin N., Schwikowski B., Ideker T. (2003). Cytoscape: A Software Environment for Integrated Models of Biomolecular Interaction Networks. Genome Res..

[52] Sedlák F., Kvasnička A., Marešová B., Brumarová R., Dobešová D., Dostálová K., Šrámková K., Pehr M., Šácha P., Friedecký D. (2024). Parallel Metabolomics and Lipidomics of a PSMA/GCPII Deficient Mouse Model Reveal Alteration of NAAG Levels and Brain Lipid Composition. ACS Chem. Neurosci..

