# Supplementary figures and images for: Aromatase Inhibitor Therapy Is Associated with Distinct Plasma Lipidomic Profiles in Postmenopausal Breast Cancer Patients
Source: Int J Mol Sci. 2026 Feb 17;27(4):1926. doi: 10.3390/ijms27041926 (PMC12940922; doi:10.3390/ijms27041926)

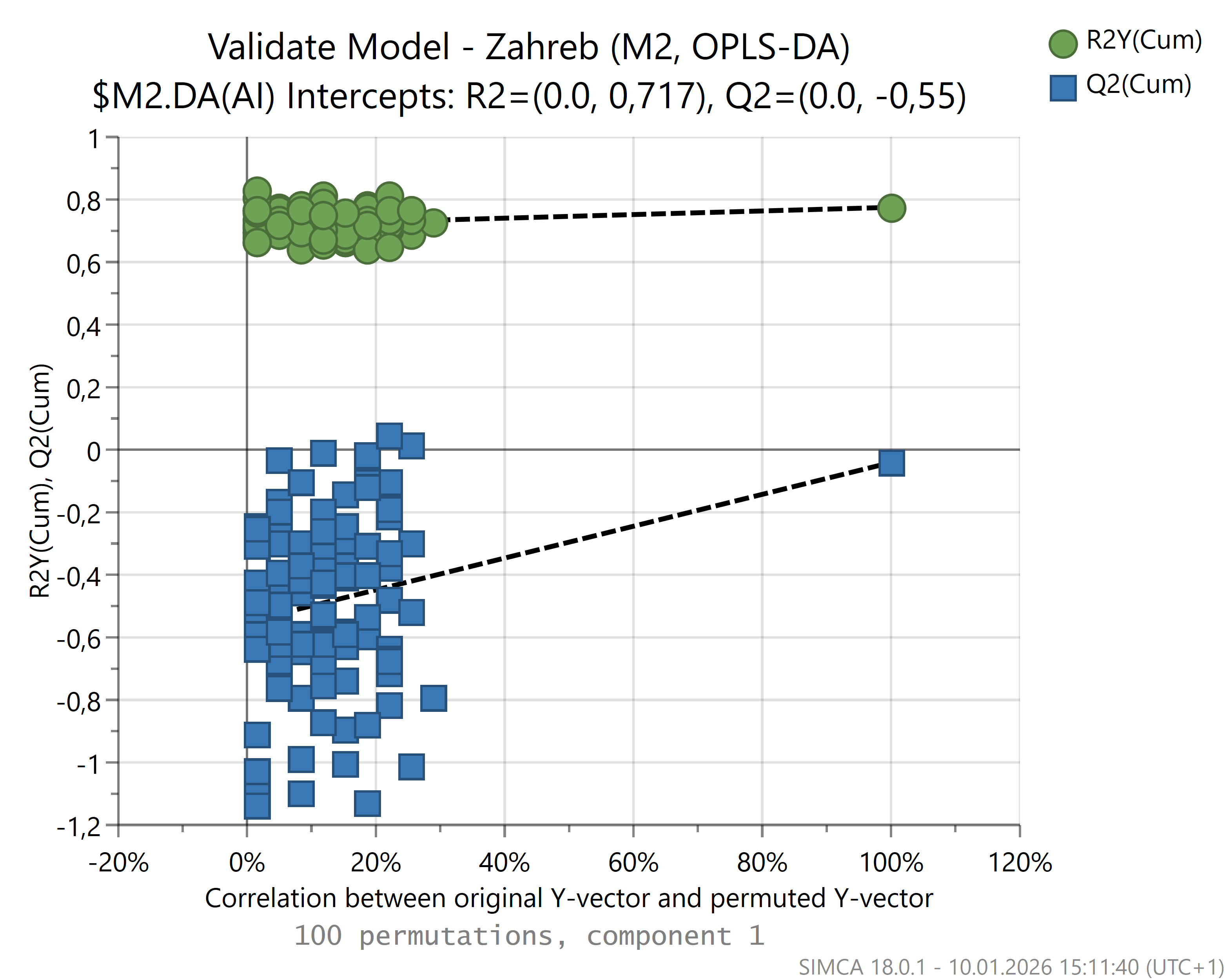

Supplement: Supplementary file 1 [file ijms-27-01926-s001.zip › Supplementary Figure S1.png]

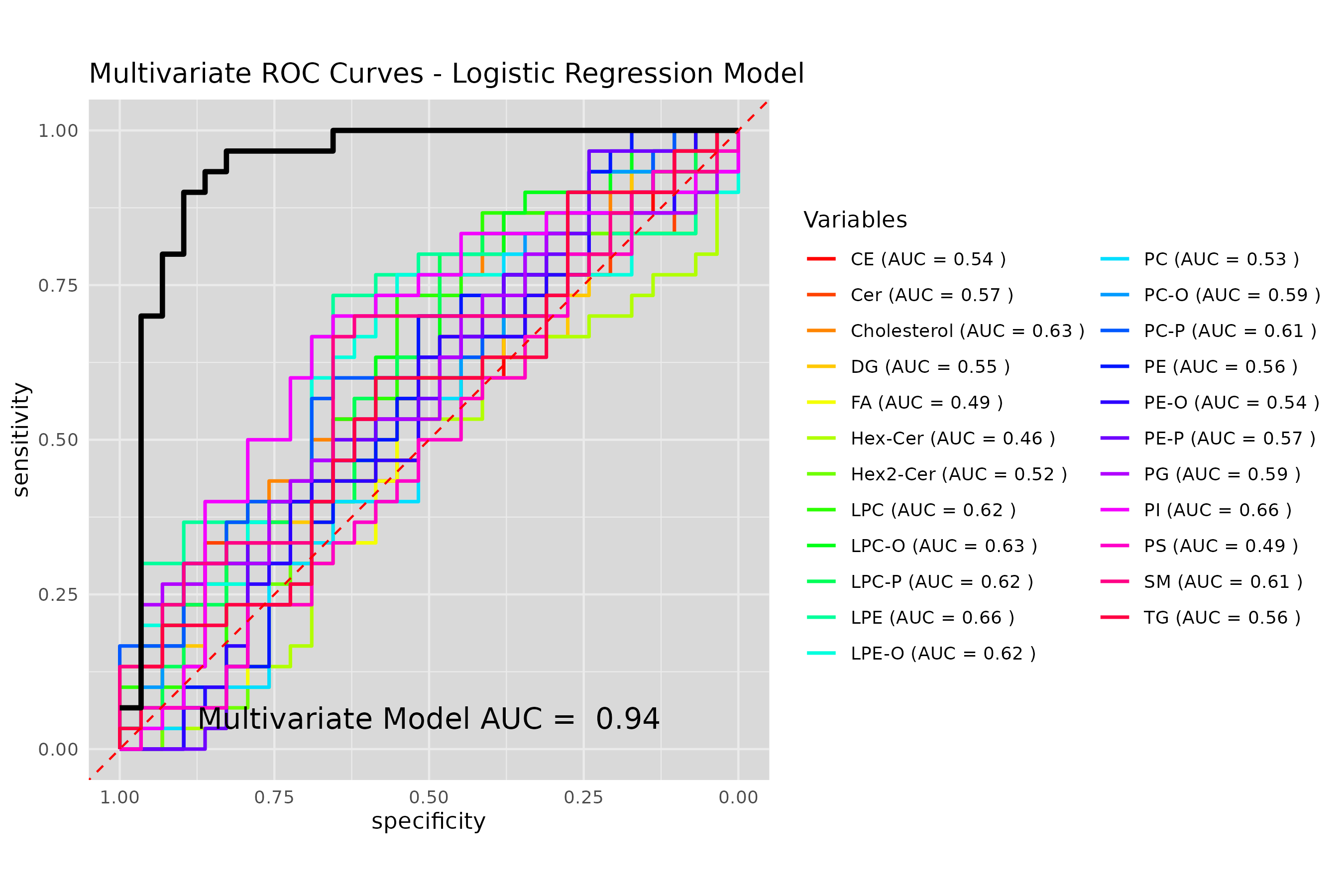

Supplement: Supplementary file 1 [file ijms-27-01926-s001.zip › Supplementary Figure S2.png]
